# Assessing the use of HL7 FHIR for implementing the FAIR guiding principles: a case study of the MIMIC-IV Emergency Department module

**DOI:** 10.1093/jamiaopen/ooae002

**Published:** 2024-01-27

**Authors:** Philip van Damme, Matthias Löbe, Nirupama Benis, Nicolette F de Keizer, Ronald Cornet

**Affiliations:** Department of Medical Informatics, Amsterdam UMC location University of Amsterdam, Amsterdam, The Netherlands; Amsterdam Public Health, Digital Health & Methodology, Amsterdam, The Netherlands; Institute for Medical Informatics, Statistics and Epidemiology (IMISE), University of Leipzig, Leipzig, Germany; Department of Medical Informatics, Amsterdam UMC location University of Amsterdam, Amsterdam, The Netherlands; Amsterdam Public Health, Digital Health & Methodology, Amsterdam, The Netherlands; Department of Medical Informatics, Amsterdam UMC location University of Amsterdam, Amsterdam, The Netherlands; Amsterdam Public Health, Methodology & Quality of Care, Amsterdam, The Netherlands; Department of Medical Informatics, Amsterdam UMC location University of Amsterdam, Amsterdam, The Netherlands; Amsterdam Public Health, Digital Health & Methodology, Amsterdam, The Netherlands

**Keywords:** FAIR guiding principles, HL7 FHIR, reusable data, MIMIC-IV

## Abstract

**Objectives:**

To provide a real-world example on how and to what extent Health Level Seven Fast Healthcare Interoperability Resources (FHIR) implements the Findable, Accessible, Interoperable, and Reusable (FAIR) guiding principles for scientific data. Additionally, presents a list of FAIR implementation choices for supporting future FAIR implementations that use FHIR.

**Materials and methods:**

A case study was conducted on the Medical Information Mart for Intensive Care-IV Emergency Department (MIMIC-ED) dataset, a deidentified clinical dataset converted into FHIR. The FAIRness of this dataset was assessed using a set of common FAIR assessment indicators.

**Results:**

The FHIR distribution of MIMIC-ED, comprising an implementation guide and demo data, was more FAIR compared to the non-FHIR distribution. The FAIRness score increased from 60 to 82 out of 95 points, a relative improvement of 37%. The most notable improvements were observed in interoperability, with a score increase from 5 to 19 out of 19 points, and reusability, with a score increase from 8 to 14 out of 24 points. A total of 14 FAIR implementation choices were identified.

**Discussion:**

Our work examined how and to what extent the FHIR standard contributes to FAIR data. Challenges arose from interpreting the FAIR assessment indicators. This study stands out for providing a real-world example of a dataset that was made more FAIR using FHIR.

**Conclusion:**

To the best of our knowledge, this is the first study that formally assessed the conformance of a FHIR dataset to the FAIR principles. FHIR improved the accessibility, interoperability, and reusability of MIMIC-ED. Future research should focus on implementing FHIR in research data infrastructures.

## Background and significance

Data that can be reused have many benefits for science, such as verifying previously published research and extending research findings through data integration.^1^^,^^2^ The FAIR (Findable, Accessible, Interoperable, and Reusable) Guiding Principles provide guidelines for improving the reusability of scientific data and have received a broad uptake in recent years.^3^ The principles accentuate that data should be findable and usable by computers, along with support for humans to reuse data. The FAIR principles encourage scientific communities to agree on several technical solutions and domain-relevant standards to improve the findability, accessibility, interoperability, and reusability of data. Previous studies have reported that communities implementing the FAIR principles would benefit from harmonization;^4^ for example, by reusing implementation choices from other communities.^5^ However, different implementations of the FAIR principles may result in data that cannot be reused across and within communities. A set of community-driven implementation choices for implementing the FAIR principles can be captured in a FAIR implementation profile.^5^ FAIR implementation profiles aim to improve the reuse of FAIR implementation choices across communities. Due to the variety of FAIR implementations and the need for a way to evaluate FAIR data, many FAIR assessment tools have been developed thus far, from questionnaires to (semi-)automated tools.^6^

In the health research community, Sinaci et al.^7^ have presented an open architecture based on the FAIR principles for managing health data in the European Union. At the core of this architecture lies Health Level Seven Fast Healthcare Interoperability Resources (HL7 FHIR), a standard that is being rapidly adopted for data exchange in healthcare.^8^ FHIR defines processes to constrain the standard’s base specification whenever needed for use in a specific context. Constraining a base FHIR resource is called profiling, and a set of constraints is called a profile. A FHIR implementation guide contains a set of rules and documentation about how FHIR should be used in a particular context. For example, 2 independent implementations of FHIR might describe different information for a patient (eg, veterinary health care vs human health care). The FHIR standard acts as a foundation where as much as is feasible is standardized. Martínez et al. have published a FHIR implementation guide (“FHIR for FAIR”) with general recommendations on how FHIR could support reusable, FAIR health data.^9^^,^^10^ Moreover, Gebreslassie et al. have presented an architecture that leverages FHIR to make the Virus Outbreak Data Network Africa more FAIR.^11^ A standard like FHIR could provide a solution for harmonizing technical implementation choices when implementing the FAIR principles. However, to date, no studies have been published that demonstrate the technical implementation of the FAIR principles using FHIR and show the degree to which FHIR can be used to implement the principles.

This article aims to enhance the general understanding and alignment of FAIR implementations through a formal assessment on how the FHIR standard contributes to FAIR data. To accomplish this, we dissect a real-world dataset, providing an illustrative example for future FAIR implementations based on FHIR. We present a case study of the Medical Information Mart for Intensive Care (MIMIC)-IV Emergency Department (ED) database. MIMIC-ED is a deidentified real-world clinical dataset that is freely available on PhysioNet, a repository for medical research data.^12^^,^^13^ MIMIC-ED is a module of MIMIC-IV^14^ and comprises data on emergency department admissions. MIMIC-IV has recently been converted into FHIR, and currently comprises an implementation guide and demo data.^15^ The objectives of this case study are as follows: (1) analyze and compare the conformance of the MIMIC-ED dataset to the FAIR principles in its original and FHIR formats; (2) based on the conformance assessments, present a list of implementation choices that provide an overview of technical choices for implementing FAIR with FHIR—aiming to support future FAIR implementations.

## Methods

**Figure 1.**
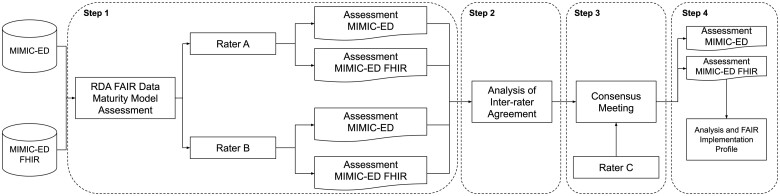
Overview of the performed case study. Abbreviations: MIMIC-ED, Medical Information Mart for Intensive Care IV Emergency Department dataset; FHIR, Fast Healthcare Interoperability Resources; RDA, Research Data Alliance; FAIR, Findable, Accessible, Interoperable, and Reusable.

A case study of the MIMIC-ED dataset was conducted to assess the extent to which the FAIR principles can be implemented using the FHIR standard. There are 2 distributions of MIMIC-ED, the original publication on PhysioNet and the FHIR distribution, which allowed for comparison. The FHIR distribution of MIMIC-ED came about as part of earlier work that converted the MIMIC-IV database into FHIR.^15^ The original distribution of MIMIC-ED consists of a web page on the PhysioNet website with metadata^12^ and a set of CSV files containing the data (version 2.0 was used for this study). The FHIR distribution comprises an FHIR implementation guide^16^ and a set of newline delimited JSON (NDJSON) files. It is assumed that the NDJSON files are loaded into a FHIR server for this assessment. The study breaks down into 4 primary steps: (1) assess, independently by 2 raters, the conformance of both the original and FHIR distributions of MIMIC-ED to the FAIR principles, using a set of standard FAIR assessment criteria; (2) analyze the inter-rater agreement; (3) reach consensus on the ratings, where needed, by consulting a third expert; and (4) analyze the final scores and compose a simplified FAIR Implementation Profile that shows how and to what extent FHIR could be used to implement the FAIR principles. Figure 1 shows a graphical overview of these steps.

### Assess data “FAIRness”

Two medical informaticians with knowledge of FHIR and FAIR independently evaluated the original and FHIR distributions of MIMIC-ED (PvD [rater A] and ML [rater B]). We used the Research Data Alliance (RDA) FAIR Data Maturity Model, a set of standard FAIR assessment criteria to measure the FAIR maturity level of a dataset.^17^ This assessment model was chosen because it allows for manual assessment, covers all FAIR principles, and is endorsed by the RDA.^17^Table 1 shows the 41 indicators, the FAIR principle each indicator relates to, and the indicator’s priority (essential: indispensable for data to be FAIR, important: contributes substantially to FAIR data, useful: nice to have). The indicators are accompanied by a document with more information and examples on how to assess each of them. Assessments were based on the definitions and descriptions in this document. Each indicator was dichotomously scored on a pass/fail basis; pass = 1 and fail = 0. Motivation for each score (ie, why did the indicator pass or fail) was noted to facilitate further analysis and clarification. This step resulted in 4 assessment reports, 1 per dataset distribution (original and FHIR) and rater (A and B).

**Table 1.** The Research Data Alliance FAIR data maturity model indicators.

FAIR principle	Indicator ID	Indicator	Priority
F1	F1-01M	Metadata are identified by a persistent identifier	Essential
F1	F1-01D	Data are identified by a persistent identifier	Essential
F1	F1-02M	Metadata are identified by a globally unique identifier	Essential
F1	F1-02D	Data are identified by a globally unique identifier	Essential
F2	F2-01M	Rich metadata are provided to allow discovery	Essential
F3	F3-01M	Metadata include the identifier for the data	Essential
F4	F4-01M	Metadata are offered in such a way that it can be harvested and indexed	Essential
A1	A1-01M	Metadata contain information to enable the user to get access to the data	Important
A1	A1-02M	Metadata can be accessed manually (ie, with human intervention)	Essential
A1	A1-02D	Data can be accessed manually (ie, with human intervention)	Essential
A1	A1-03M	Metadata identifier resolves to a metadata record	Essential
A1	A1-03D	Data identifier resolves to a digital object	Essential
A1	A1-04M	Metadata are accessed through standardized protocol	Essential
A1	A1-04D	Data are accessible through standardized protocol	Essential
A1	A1-05D	Data can be accessed automatically (ie, by a computer program)	Important
A1.1	A1.1-01M	Metadata are accessible through a free access protocol	Essential
A1.1	A1.1-01D	Data are accessible through a free access protocol	Important
A1.2	A1.2-01D	Data are accessible through an access protocol that supports authentication and authorization	Useful
A2	A2-01M	Metadata are guaranteed to remain available after data are no longer available	Essential
I1	I1-01M	Metadata use knowledge representation expressed in standardized format	Important
I1	I1-01D	Data use knowledge representation expressed in standardized format	Important
I1	I1-02M	Metadata use machine-understandable knowledge representation	Important
I1	I1-02D	Data use machine-understandable knowledge representation	Important
I2	I2-01M	Metadata use FAIR-compliant vocabularies	Important
I2	I2-01D	Data use FAIR-compliant vocabularies	Useful
I3	I3-01M	Metadata include references to other metadata	Important
I3	I3-01D	Data include references to other data	Useful
I3	I3-02M	Metadata include references to other data	Useful
I3	I3-02D	Data include qualified references to other data	Useful
I3	I3-03M	Metadata include qualified references to other metadata	Important
I3	I3-04M	Metadata include qualified references to other data	Useful
R1	R1-01M	Plurality of accurate and relevant attributes are provided to allow reuse	Essential
R1.1	R1.1-01M	Metadata include information about the license under which the data can be reused	Essential
R1.1	R1.1-02M	Metadata refer to a standard reuse license	Important
R1.1	R1.1-03M	Metadata refer to a machine-understandable reuse license	Important
R1.2	R1.2-01M	Metadata include provenance information according to community-specific standards	Important
R1.2	R1.2-02M	Metadata include provenance information according to a cross-community language	Useful
R1.3	R1.3-01M	Metadata comply with a community standard	Essential
R1.3	R1.3-01D	Data comply with a community standard	Essential
R1.3	R1.3-02M	Metadata are expressed in compliance with a machine-understandable community standard	Essential
R1.3	R1.3-02D	Data are expressed in compliance with a machine-understandable community standard	Important

Abbreviations: D, data; FAIR, Findable, Accessible, Interoperable, and Reusable; M, metadata; RDA, Research Data Alliance (RDA). Adapted from RDA.^17^

### Inter-rater agreement

Inter-rater agreement is defined as the degree of agreement between the 2 raters that scored the 41 indicators for each distribution of the dataset. We measured the inter-rater agreement after both raters independently rated MIMIC-ED in the original and FHIR formats. Additionally, we noted any discrepancies in how the indicators were interpreted by the raters, which were addressed during the consensus step. First, we calculated percent agreement as a simple index for agreement between the 2 raters. We then calculated Cohen’s kappa as a chance-corrected index for inter-rater agreement.^18^^,^^19^ Kappa values were interpreted using the classification published by Landis and Koch.^20^ A kappa value between 0.0 and 0.2 means slight agreement, 0.21 and 0.4 fair agreement, 0.41 and 0.6 moderate agreement, 0.61 and 0.8 substantial agreement, and >0.8 almost perfect agreement.

### Reaching consensus

A consensus meeting was held to discuss differences in indicator ratings between the same distribution of MIMIC-ED. Discrepancies occurred when the same indicator for the same distribution of the dataset was scored differently. Although the RDA FAIR data maturity model is intended such that indicators are understood similarly, different interpretations could still occur. To reach a consensus, the raters first agreed on an indicator’s interpretation by referencing the specification and guidelines document.^17^ Then, they discussed how the indicator applied to the dataset. If a discrepancy could not be resolved, a third rater C, with expert knowledge of FAIR data (NB) made the final decision on the indicator’s score (pass or fail). This step resulted in 1 final assessment per MIMIC-ED distribution.

For example, there could be disagreement on indicator F1-01M (“Metadata is identified by a persistent identifier,” Table 1) between the assessments of the original distribution of MIMIC-ED. The specification and guidelines document states that the persistence of an identifier should be determined based on the policy or reputation of the organization that manages the identifier—which could clarify the researcher’s interpretation. There should then be a discussion about what the metadata identifier is, which organization manages it, and whether or not that organization has a policy or reputation that assures persistence. The 2 raters, A and B, can then decide on an agreed score. Should this discussion not resolve the discrepancy, rater C would decide the final score of this indicator.

### Analysis and FAIR implementation profile

We quantitatively and qualitatively analyzed the differences between the original and FHIR distributions of MIMIC-ED based on the final assessments. To quantify the level of FAIRness of and between both distributions, we calculated the sum of the indicator scores. To take the priority of an indicator into account, we first multiplied each indicator score (1’s and 0’s) with a weight corresponding to its priority. Essential indicators were assigned a weight of 3, important indicators a weight of 2, and useful indicators a weight of 1. With 41 indicators, 20 essential, 14 important, and 7 useful, an entirely FAIR dataset would receive a summative score of 95. These are our adaptations to the framework, which allows flexibility in the scoring mechanisms. The qualitative analysis encompassed summarizing the motivation(s) for the scored indicators; for example, a reason for passing indicator F1-01M could be the presence of a Digital Object Identifier.

A FAIR Implementation Profile describes a set of technical solutions to implement the FAIR principles.^5^ Based on our MIMIC-ED case study, we used the FAIR Implementation Profile mini questionnaire^21^ to list the implementation choices for implementing the principles with the FHIR standard. In essence, the questionnaire reveals how a FAIR principle was implemented. The assessment indicators indicate whether the principle was applied. Table 2 shows this questionnaire. Each question refers to a FAIR principle, corresponding to the principles and indicators listed in Table 1. We used the motivations for the indicator scores to fill out this questionnaire and create an overview of characteristics or components of the FHIR standard that contribute to FAIR data.

**Table 2.** The FAIR Implementation Profile mini questionnaire.

ID/FAIR principle	Question
F1-M	What globally unique, persistent, resolvable identifiers do you use for metadata records?
F1-D	What globally unique, persistent, resolvable identifiers do you use for datasets?
F2	Which metadata schemas do you use for findability?
F3	What is the technology that links the persistent identifiers of your data to the metadata description?
F4-M	In which search engines are your metadata records indexed?
F4-D	In which search engines are your datasets indexed?
A1.1-M	Which standardized communication protocol do you use for metadata records?
A1.1-D	Which standardized communication protocol do you use for datasets?
A1.2-M	Which authentication and authorization technique do you use for metadata records?
A1.2-D	Which authentication and authorization technique do you use for datasets?
A2	Which metadata longevity plan do you use?
I1-M	Which knowledge representation languages (allowing machine interoperation) do you use for metadata records?
I1-D	Which knowledge representation languages (allowing machine interoperation) do you use for datasets?
I2-M	Which structured vocabularies do you use to annotate your metadata records?
I2-D	Which structured vocabularies do you use to encode your datasets?
I3-M	Which models, schema(s) do you use for your metadata records?
I3-D	Which models, schema(s) do you use for your datasets?
R1.1-M	Which usage license do you use for your metadata records?
R1.1-D	Which usage license do you use for your datasets?
R1.2-M	Which metadata schemas do you use for describing the provenance of your metadata records?
R1.2-D	Which metadata schemas do you use for describing the provenance of your datasets?

Answers provide the chosen technical solutions to implement the FAIR principles. Abbreviations: D, data; FAIR, Findable, Accessible, Interoperable, and Reusable; Metadata (M). Adapted from GO FAIR.^21^

## Results

Both raters gave different scores to 10 indicators of the original distribution and 9 indicators of the FHIR distribution after independently assessing MIMIC-ED’s FAIRness. A discussion between raters A and B in the consensus meeting resolved 2 and 5 discrepancies for the original and FHIR distributions, respectively. The remaining differences, a total of 12, were assessed by rater C to reach a consensus on all indicators. Discrepancies were found for indicators on identifiers, metadata, knowledge representation formats, references, and license information. Percent agreement for the original and FHIR distributions of MIMIC-ED was 76% and 78%, respectively. Kappa values were 0.51 95% CI [0.26, 0.75] (original distribution, moderate agreement) and 0.38 95% CI [0.097, 0.66] (FHIR distribution, fair agreement).

After multiplying each indicator’s score with its corresponding weight, the original distribution of MIMIC-ED received a total score of 60 out of 95 points, which means the dataset is 63% FAIR according to our assessment. On the other hand, the FHIR distribution received a total score of 82 out of 95 points or 86% FAIR. Thus, we found that the FHIR distribution of MIMIC-ED is 23 percentage points (a relative change of 37%) more FAIR than the original distribution. The FHIR distribution received the same score as the original distribution for 23 indicators. Fifteen indicators of the FHIR distribution received a higher score. Finally, 3 indicators of the FHIR distribution were rated lower than the original distribution. Table 3 shows all scores for both distributions and raters and for which indicators a consensus was reached by a discussion between raters A and B or whether the indicator was assessed by rater C. The full assessment reports with qualitative comments on each indicator are included as Supplementary Files.

**Table 3.** Assessment results of the original and FHIR distributions of the MIMIC-ED dataset from the RDA FAIR Data Maturity Model indicators (Table 1).

	MIMIC-ED score (original)				MIMIC-ED score (FHIR)				Final weighted scores (after consensus)	
Indicator ID	Rater A	Rater B	AB	C	Rater A	Rater B	AB	C	Original	FHIR
F1-01M	1	1			1	1			3	3
F1-01D	1	1			0	1		✓	3	0
F1-02M	1	1			1	1			3	3
F1-02D	1	1			0	1		✓	3	3
F2-01M	1	1			1	1			3	3
F3-01M	1	0		✓	0	1	✓		0	3
F4-01M	1	0		✓	1	1			3	3
									**18/21**	**18/21**
A1-01M	1	1			1	1			2	2
A1-02M	1	1			1	1			3	3
A1-02D	1	1			1	1			3	3
A1-03M	1	1			1	1			3	3
A1-03D	1	1			1	1			3	3
A1-04M	1	1			1	1			3	3
A1-04D	1	1			1	1			3	3
A1-05D	1	0	✓		1	1			0	2
A1.1-01M	1	1			1	1			3	3
A1.1-01D	1	1			1	1			2	2
A1.2-01D	1	1			1	1			1	1
A2-01M	1	1			0	1	✓		3	3
									**29/31**	**31/31**
I1-01M	0	0			1	1			0	2
I1-01D	1	0		✓	1	1			0	2
I1-02M	0	0			1	1			0	2
I1-02D	1	0	✓		1	1			0	2
I2-01M	0	0			1	1			0	2
I2-01D	0	0			0	1		✓	0	1
I3-01M	1	0		✓	1	1			2	2
I3-01D	0	0			0	1	✓		0	1
I3-02M	1	1			1	1			1	1
I3-02D	0	0			0	1	✓		0	1
I3-03M	1	0		✓	1	1			2	2
I3-04M	1	0		✓	0	1	✓		0	1
									**5/19**	**19/19**
R1-01M	1	1			1	1			3	3
R1.1-01M	1	1			0	0			3	0
R1.1-02M	1	1			0	0			2	0
R1.1-03M	0	0			0	0			0	0
R1.2-01M	0	0			0	1		✓	0	0
R1.2-02M	0	0			0	0			0	0
R1.3-01M	1	0		✓	1	1			0	3
R1.3-01D	0	1		✓	1	1			0	3
R1.3-02M	0	0			1	1			0	3
R1.3-02D	0	0			1	1			0	2
									**8/24**	**14/24**
									**60/95**	**82/95**

Bold values indicate sum scores. Abbreviations: AB, consensus reached by discussion between rater A and B; C, consensus reached by rater C; FAIR, Findable, Accessible, Interoperable, and Reusable; FHIR, Fast Healthcare Interoperability Resources; MIMIC-ED, Medical Information Mart for Intensive Care IV Emergency Department dataset; RDA, Research Data Alliance.

Table 4 shows the FAIR implementation choices of MIMIC-ED FHIR (ie, the answers to the FAIR Implementation Profile mini questionnaire) with references to relevant parts of the FHIR specification. Question A1.2-M (“Which authentication & authorization technique do you use for metadata records?”) did not apply to MIMIC-ED, as the metadata is publicly available and does not require any form of access control. For all other questions, Table 4 indicates whether the underlying principle of that question was implemented, not implemented, or partially implemented.

**Table 4.** Answers to the FAIR Implementation Profile mini questionnaire for MIMIC-ED.

Question ID	Keyword	Implemented?	Implementation choice MIMIC-ED FHIR
F1-M	Identifiers	✓	Canonical identifier (absolute URI) of the implementation guide^22^
F1-D	Identifiers	Partially	Only globally unique identifiers (resource ID + server URL)^23^
F2	Metadata schemas	✓	ImplementationGuide and CapabilityStatement resource structure definitions^24^^,^^25^
F3	Identifier linking	✗	Data identifiers are not persistent
F4-M	Search engine indexing	✓	The implementation guide can be found by general search engines (eg, Google)
F4-D	Search engine indexing	✗	Not indexed because the data is not publicly available (credentialed access PhysioNet)
A1.1-M	Communication protocol	✓	HTTP (the published implementation guide as HTML files and the FHIR RESTful API)^26^
A1.1-D	Communication protocol	✓	HTTP (the FHIR RESTful API)^26^
A1.2-M	Authentication and authorization	N/A	No authentication or authorization for accessing the metadata (implementation guide)
A1.2-D	Authentication and authorization	✓	Users that load the data into their FHIR server(s) should ensure the use of TLS^27^
A2	Metadata longevity	✓	The implementation guide is hosted on the official MIMIC website maintained by MIT^16^
I1-M	Knowledge representation	✓	JSON, XML, or RDF (Turtle)^28^
I1-D	Knowledge representation	✓	NDJSON (for bulk import) and once on a server JSON, XML, or RDF (Turtle)^28^
I2-M	Vocabularies	✓	All vocabularies (in structure definitions) follow the FHIR terminologies specification^29^
I2-D	Vocabularies	✓	All vocabularies (in data resources) follow the FHIR terminologies specification^29^
I3-M	Metadata model	✓	ImplementationGuide, StructureDefinition, ValueSet, and CodeSystem resource structure definitions^24^^,^^30–32^
I3-D	Data model	✓	Resource profiles as described in structure definitions^33^
R1.1-M	License	✗	License information is not included in the FHIR distribution of MIMIC-ED
R1.1-D	License	✗	License information is not included in the FHIR distribution of MIMIC-ED
R1.2-M	Provenance	✗	Provenance information is not included in the FHIR distribution of MIMIC-ED
R1.2-D	Provenance	✗	Provenance information is not included in the FHIR distribution of MIMIC-ED

Abbreviations: API, application programming interface; FAIR, Findable, Accessible, Interoperable, and Reusable; FHIR, Fast Healthcare Interoperability Resources; HTML, HyperText Markup Language; (ND)JSON, (Newline Delimited) JavaScript Object Notation; MIMIC-ED, Medical Information Mart for Intensive Care IV Emergency Department dataset; MIT, Massachusetts Institute of Technology; REST, REpresentational State Transfer; RDF, Resource Description Framework; TLS, Transport Layer Security; URI, Uniform Resource Identifier; XML, Extensible Markup Language.

## Discussion

This work aimed to examine how and to what extent the FHIR standard can be used to implement the FAIR principles. Additionally, it seeks to support (future) FAIR implementations of communities that plan on or are using FHIR to make their data more FAIR. By performing a case study on MIMIC-ED, a module of a large deidentified real-world clinical dataset that has been converted into FHIR, we found that FHIR improved the FAIRness of this dataset from 60 to 82 out of 95 points compared to its original distribution. The most significant improvements were achieved in interoperability (5–19 out of 19 points) and reusability (8–14 points out of 24 points). To implement the FAIR principles, communities must make multiple technical decisions, which can be challenging since many available solutions exist. We quantified these choices for the FHIR distribution of MIMIC-ED to further aid in harmonizing FAIR implementation choices between communities.

Our results support the hypothesis that FHIR could be used to make data more FAIR. While we did observe an overall improvement in FAIRness, the indicators F1-01D (“data is identified by a persistent identifier”), R1.1-01M (“metadata includes information about the license under which the data can be reused”), and R1.1-02M (“metadata refers to a standard reuse license”) received a lower score for FHIR than for the original dataset. The original dataset has a persistent identifier and is available for download on PhysioNet to credentialed users that signed a data use agreement. Because the data are not openly available, users must load the FHIR data into their own FHIR server; therefore, the FHIR data identifiers are not persistent. This indicator would pass if the data were served through only 1 server, as FHIR resource IDs are persistent within the context of a server. Utilizing the Identifier element within a FHIR resource allows for the inclusion of business identifiers, such as social security numbers. This facilitates the identification of resources across FHIR servers that pertain to the same real-world entity. While all identifiers in MIMIC-ED were substituted with randomized surrogates, the Identifier element could be valuable in other FAIR data use cases. The MIMIC-ED FHIR metadata did not contain license information. Since PhysioNet handled license agreements and data distribution, license information was not captured in FHIR. In addition, PhysioNet follows many best practices for data management, which makes the original distribution of MIMIC-ED score well on findability and accessibility. Those datasets or repositories not yet following such best practices may benefit even more from FHIR.

Several noteworthy improvements can be observed in comparing the original dataset in CSV format to the FHIR distribution. First, the original dataset only offers human-readable descriptions in its metadata on the PhysioNet website. The FHIR distribution, however, significantly improves this by incorporating machine-readable metadata through StructureDefinitions (profiles) in the implementation guide. Second, although the original CSV dataset follows a structured format, it lacks a standard data model. In contrast, the FHIR distribution adheres to the models specified in the MIMIC FHIR profiles, and the data are available in JSON format. Finally, the original dataset contains a human-readable license description; this information was not included in the FHIR distribution.

The indicators related to provenance information (R1.2-01M and R1.2-02M) did not pass for either distribution of MIMIC-ED. This was due to MIMIC-IV (and MIMIC-ED) not including provenance information; therefore, the FHIR distribution did not include provenance information either. It should be noted that FHIR provides mechanisms for capturing provenance information about resources. First, FHIR servers store a distribution history of every resource it contains. Secondly, every resource contains some provenance information that conforms to a common logical model. This logical model defines a pattern based on the 5 Ws—Who, What, When, Where, Why.^34^ Notably, every FHIR resource has an optional “meta” element that contains metadata about a resource. The element “Meta.source” can reference the resource’s source system; the “Meta.tag” element can relate a resource to certain processes or workflows. Finally, FHIR defines a Provenance resource that can be used to supplement provenance information that was already defined in other resources.^35^

To the best of our knowledge, this is the first study that formally assessed the use of the FHIR standard for implementing the FAIR principles. Our findings are consistent with previous studies that have made recommendations on how FHIR can advance the FAIRness of health data.^9^^,^^11^ Based on our results, FHIR seems to have the largest positive impact on data interoperability and reusability. A FAIR implementation based on FHIR would likely benefit from a combination of other solutions or standards, especially if the solution provided by FHIR does not meet specific community requirements. For example, in the case of MIMIC-ED, PhysioNet would be the preferred provider for handling data access requests and assigning a persistent identifier to the dataset. Furthermore, building and enforcing implementation guides are essential for (re)usable FAIR implementations based on FHIR, notably for capturing data-independent metadata. In light of interoperability between FAIR communities and implementations, compatibility between FHIR profiles and distributions should be taken into consideration from an early stage. Methods such as the “FHIR Interoperability Table” presented by Kramer and Moesel,^36^ could be useful to identify compatibility issues.

Our work has some limitations. First, evaluators can interpret the RDA FAIR Data Maturity Model indicators differently despite their accompanying guidelines. This conclusion is reflected by our obtained Kappa values, indicating moderate to fair agreement. We intended to avoid misinterpretations as much as possible by assigning a third rater to indicators without a consensus and by only using definitions listed in the maturity model documentation. Secondly, as this is a case study, our assessment applies to only one dataset. There are various (other) scenarios to which the FAIR principles can be applied, from single datasets like MIMIC-ED to (inter)national research infrastructures. Although the identified FAIR implementation choices appear to be generalizable, the assessment results cannot be extrapolated without additional analysis. In spite of this limitation, we find MIMIC-IV to be a compelling example due to its extensive utilization in research studies,^14^ its availability, and its metadata. Consequently, we believe that FHIR’s potential to improve data FAIRness may be even greater for datasets that do not already follow best practices, as opposed to those on PhysioNet.

Our work also has several strengths. The FAIR principles are broadly adopted but are also known to be misinterpreted or misunderstood.^37^ This work provides a concrete example of an FHIR dataset and shows which aspects of FAIR it implements and how, thus, serving as a reference for future FAIR implementations that use FHIR. Additionally, by adopting the FAIR Implementation Profile mini questionnaire, our work fits into existing efforts to spread the reuse of implementation choices between FAIR communities. We hope that by providing such real-world examples, the FAIR principles can be easier understood and implemented.

The FHIR standard offers technical solutions for the FAIR principles that make harmonization between FAIR implementations easier. Naturally, FHIR is not suited for all types of data and will not be the end-all solution for FAIR data. The HL7 Biomedical Research & Regulations working group is driving ongoing initiatives that specifically aim to explore the application of FHIR in clinical research use cases.^38^ In order to evaluate the use of FHIR for FAIR data on a larger scale, including the FAIRness of multiple FHIR endpoints and implementation guides, further research is needed. In addition, future research should attempt to extend generally applicable clarifications of FAIR principles (eg, the meaning of indicator I2-01MD “FAIR-compliant vocabularies” [Table 1] could be specified using work such as that of Xu et al.^39^) Finally, we intend to convert the FHIR implementation choices of MIMIC-ED into a machine-readable FAIR implementation profile to enable reuse beyond the target audience of this paper.

## Conclusions

This is the first study that formally assessed the use of FHIR for implementing the FAIR principles. Our case study of MIMIC-ED showed that the conversion into FHIR enhanced the accessibility, interoperability, and reusability of the dataset. We presented a list of FAIR implementation choices that should contribute to further harmonizing FAIR communities that aim to use FHIR. Future research should focus on implementing FHIR in research data infrastructures for FAIR health data.

## Supplementary Material

ooae002_Supplementary_Data

## Data Availability

The assessment reports that contain all the scores and qualitative comments on each indicator are included as supplementary files. The full dataset is available on Dryad: https://doi.org/10.5061/dryad.1jwstqk10.
