# Supplementary material for: Assessing the use of HL7 FHIR for implementing the FAIR guiding principles: a case study of the MIMIC-IV Emergency Department module
Source: JAMIA Open. 2024 Jan 27;7(1):ooae002. doi: 10.1093/jamiaopen/ooae002 (PMC10822118; doi:10.1093/jamiaopen/ooae002)
Supplement: ooae002_Supplementary_Data [file ooae002_supplementary_data.zip › Supplementary File 1 - PhysioNet Distribution Indicator Scores and Qualitative Comments.docx]

| **Indicator ID** | **Score Rater A** | **Motivation Rater A** | **Score Rater B** | **Motivation Rater B** | **Motivation Rater C** | **Final Score** |
| --- | --- | --- | --- | --- | --- | --- |
| F1-01M | 1 | DOI, the metadata is included on the same HTML page (text). | 1 | DOI |  | 1 |
| F1-01D | 1 | Link to a ZIP file to download the data. | 1 | I assume it is from the structure of the provided URLs (containing 'static' in the URL). No DOI. |  | 1 |
| F1-02M | 1 | As F1-01M | 1 | Yes, only a single resource is addressed. |  | 1 |
| F1-02D | 1 | As F1-01D | 1 | Yes, only a single resource is addressed. |  | 1 |
| F2-01M | 1 | A detailed decription of the data(items) that allow users to know what the data contains and is about, also license and access information. | 1 | Readable for humans, no discovery like DCAT. The metadata set is minimal and could be improved. |  | 1 |
| F3-01M | 1 | The link to the data is included on the HTML page. | 0 | Only a link to a ZIP file. | This indicator implies that the data is assigned an identifier and can be retrieved with that identifier. This is not the case here. | 0 |
| F4-01M | 1 | The page is indexed by all general search engines (e.g., Google). | 0 | Only text-based. PhysioNet's own search seems to use additional structured data elements that are not usable by other engines. | While the format presented is not ideal for machine findability, it does fullfill the indicator's requirements. | 1 |
| A1-01M | 1 | The page includes instructions on how to access the data, the instructions are not machine-readable but are clear for humuns to follow. | 1 | Data access policy is well explained. |  | 1 |
| A1-02M | 1 | Publicly available HTML page. | 1 | Yes |  | 1 |
| A1-02D | 1 | Need for a PhysioNet credentialed account and agreeing with a data-use policy. After that, the data is available to download. | 1 | Yes |  | 1 |
| A1-03M | 1 | Resolvable DOI to HTML page. | 1 | Yes |  | 1 |
| A1-03D | 1 | Resolvable URL to downloadable ZIP file (once data access is granted). | 1 | Yes |  | 1 |
| A1-04M | 1 | HTTP | 1 | Yes, HTTP. |  | 1 |
| A1-04D | 1 | HTTP | 1 | Yes, HTTP. |  | 1 |
| A1-05D | 1 | Using HTTP GET the ZIP files can be retrieved, but not the ready-to-use data. | 0 | ZIP files can be downloaded. Access to data means being able to interact with/process the data directly. |  | 0 |
| A1.1-01M | 1 | HTTP | 1 |  |  | 1 |
| A1.1-01D | 1 | HTTP | 1 |  |  | 1 |
| A1.2-01D | 1 | Using the HTTP request header the data can be accessed using a PhysioNet username and password. | 1 | Yes, but manually. HTTP does offer this, but that makes this indicator trivial to pass. |  | 1 |
| A2-01M | 1 | The PhysioNet page stays online even when the data is no longer available. | 1 | Seems to be a PhysioNet policy, older versions are accessible. |  | 1 |
| I1-01M | 0 | The metadata is only available as free-text and is not standardized (the page is HTML but not structured). | 0 | HTML? Potential discussion on what is structured (are headings structured? for example). In general, people would use HTML pages with sections/headings etc. If this would be 1 (given the page), what would a page look like that scores 0? |  | 0 |
| I1-01D | 1 | CSV (which is mentioned by Fairsharing). | 0 | CSV is a standard in some way. I argue that CSV is not appropriate for the health data domain. | CSV is a structured format but it is used to represent the data and not the data model which is not explicitly available. | 0 |
| I1-02M | 0 | No, only free text. | 0 |  |  | 0 |
| I1-02D | 1 | No (standardized) data model used. | 0 |  |  | 0 |
| I2-01M | 0 | No vocabularies are used. | 0 | No |  | 0 |
| I2-01D | 0 | The data does include codes (e.g. ICD-9/10), but these are not documented nor resolvable. | 0 | Partially, when using medical terminologies like LOINC. |  | 0 |
| I3-01M | 1 | There are links to the authors' profile pages on PhysioNet that contain more information about that particular author. | 0 | Partially, e.g. authors are linked. There should be an option to traverse between metadata objects (just one metadata element that links to another metadata object/element is not sufficient). | Ideally, all the metadata that can be linked to external sources should be linked; however, linking authors is a start. | 1 |
| I3-01D | 0 | The data does not contain references to other data. | 0 | Partially, foreign keys constraints. There are no references from this dataset to other datasets. |  | 0 |
| I3-02M | 1 | Reference to MIMIC-IV. | 1 | Because of the boxes that link to the previous versions (on the PhysioNet page). |  | 1 |
| I3-02D | 0 | No (qualified; with the relationship specified) references to other data. | 0 | No |  | 0 |
| I3-03M | 1 | The references in the metadata to other metadata (the author pages), does state that the reference is about 'author info', author refers to the author of the dataset. | 0 | No | Only one type of metadata is referenced but it does have some information on the type of reference. | 1 |
| I3-04M | 1 | The metadata specify that MIMIC-ED is a module of MIMIC-IV. | 0 |  | Although there is a brief mention of another dataset, the representation of the relationship is not structured and hence not easy to find. | 0 |
| R1-01M | 1 | PsyioNet has author guidelines for metadata that describe which elements should be included. | 1 |  |  | 1 |
| R1.1-01M | 1 | Metadata includes a link to the license. | 1 |  |  | 1 |
| R1.1-02M | 1 | I would argue that PhysioNet is large and influencial enough that their license can be considered standard. | 1 | Consortia-wide. The license is elaborate, consistent throughout physionet, and there is not much health data publicly available (for obvious reasons), so this license seems appropriate. |  | 1 |
| R1.1-03M | 0 | Licence info is not machine readable (free text). | 0 | No. |  | 0 |
| R1.2-01M | 0 | The MIMIC-ED page describes the provenance of the data, but in the PhysioNet Author Guidelines there is no mention of provenance metadata. So, I would not say the provenance information follows a community guideline. | 0 | Versions. |  | 0 |
| R1.2-02M | 0 | No provenance information that follow cross-community languages. | 0 | No. |  | 0 |
| R1.3-01M | 1 | The metadata complies with PhysioNet's guidelines for metadata. | 0 | HTML. | A community standard is usually accepted by various institutions and groups within a community. Since the PhysioNet Author Guidelines appear to be specifc to PhysioNet and not necessarily endorsed by the community as a whole it does not qualify. | 0 |
| R1.3-01D | 0 | Other than being available as CSV files, the data does not follow community standards. | 1 | CSV. Data is reusable if it not too hard to parse it and reuse it for my own program. For nteroperability, this is a different case. The quality of the CSV files matter. | While the CSV might be well structured, it does not seem to follow a data model that is accepted by the community as a whole. | 0 |
| R1.3-02M | 0 | The metadata standard (description on the PhysioNet website) is only available as free text. | 0 | No. |  | 0 |
| R1.3-02D | 0 | The description of CSV would be machine-understandable, but the data does not follow any other standards. | 0 | Limited. |  | 0 |
