# Supplementary material for: Assessing the use of HL7 FHIR for implementing the FAIR guiding principles: a case study of the MIMIC-IV Emergency Department module
Source: JAMIA Open. 2024 Jan 27;7(1):ooae002. doi: 10.1093/jamiaopen/ooae002 (PMC10822118; doi:10.1093/jamiaopen/ooae002)
Supplement: ooae002_Supplementary_Data [file ooae002_supplementary_data.zip › Supplementary File 2 - FHIR Distribution Indicator Scores and Qualitative Comments.docx]

| **Indicator ID** | **Score Rater A** | **Motivation Rater A** | **Score Rater B** | **Motivation Rater B** | **Motivation Rater C** | **Final Score** |
| --- | --- | --- | --- | --- | --- | --- |
| F1-01M | 1 | The metadata is available as part of the implementation guide, which has a persistent identifier. | 1 |  |  | 1 |
| F1-01D | 0 | Currently, the data is not available through a single FHIR server that can serve persistent identifiers (the data is hosted on PhysioNet and available for users to load onto their own server). | 1 |  | Persistence indicates that the data can be accessed or identified with that identifier for a certain amount of time. However, there does not seem to be a requirement to formally declare the expected persistence of a FHIR resource id by the creator of a FHIR server for the MIMIC-ED data (multiple servers could point to the same data). In all the data related indicators for FHIR there is an assumption of there being a FHIR server with the data on it. | 0 |
| F1-02M | 1 | Yes, the implementation guide identifier (and profile identifiers) are globally unique. | 1 |  |  | 1 |
| F1-02D | 0 | Same as F1-01D. | 1 | Only if there is a FHIR server. | While there could be different identifiers for the same data, if there are multiple implementations of FHIR servers with MIMIC-ED data, each identifier is still globally unique. In all the data related indicators for FHIR there is an assumption of there being a FHIR server with the data on it. | 1 |
| F2-01M | 1 | The IG provides a full description of the data and includes tutorials on how to access the data. | 1 |  |  | 1 |
| F3-01M | 0 | The identifier for the data could be different (e.g. when someone sets up their own FHIR server, the data would be downloadable from PhysioNet, just like the demo data already is). However, a FHIR server's CapabilityStatement would include links to the data. | 1 | Only if there is a FHIR server. |  | 1 |
| F4-01M | 1 | The metadata/implementation guide can be indexed by general search engines (e.g. Google). | 1 |  |  | 1 |
| A1-01M | 1 | The implementation guide contains tutorials on how to access/load the data. | 1 | Tutorials. |  | 1 |
| A1-02M | 1 | The implementation guide can be accessed like any other website. | 1 |  |  | 1 |
| A1-02D | 1 | The data can be loaded onto a FHIR server and/or be read directly from NDJSON files. | 1 | Only if there is a FHIR server. |  | 1 |
| A1-03M | 1 | Yes, the URL resolves to the implementation guide index page. | 1 |  |  | 1 |
| A1-03D | 1 | Once a FHIR server is set up, the server URL follow standard HTTP/REST principles. | 1 | Only if there is a FHIR server. |  | 1 |
| A1-04M | 1 | HTTP (webpage or FHIR server API). | 1 |  |  | 1 |
| A1-04D | 1 | HTTP | 1 | Only if there is a FHIR server. |  | 1 |
| A1-05D | 1 | Yes, using the FHIR REST API (HTTP). | 1 | Only if there is a FHIR server. |  | 1 |
| A1.1-01M | 1 | HTTP | 1 |  |  | 1 |
| A1.1-01D | 1 | FHIR REST API | 1 | Only if there is a FHIR server. |  | 1 |
| A1.2-01D | 1 | FHIR REST API | 1 |  |  | 1 |
| A2-01M | 0 | The implementation guide remains online when the data (or FHIR server) is no longer available. Data and metadata are separated. | 1 |  |  | 1 |
| I1-01M | 1 | Either JSON, XML, or RDF (Turtle). | 1 |  |  | 1 |
| I1-01D | 1 | Either JSON, XML, or RDF (Turtle). | 1 | Only if there is a FHIR server. |  | 1 |
| I1-02M | 1 | Using the FHIR data model, whereas the MIMIC-ED profiles are StructureDefinition resources. | 1 |  |  | 1 |
| I1-02D | 1 | Using the FHIR data model conforming to specific MIMIC-ED profiles. | 1 |  |  | 1 |
| I2-01M | 1 | All the vocabularies that are used are resolvable and use globally unique and persistent identifiers. | 1 |  |  | 1 |
| I2-01D | 0 | Most vocabularies are, but MIMIC-ED contains ICD-9 and ICD-10 codes that do not reference the actual ICD code systems, but instead use custom code systems (for data consistency purposes). | 1 | Only if there is a FHIR server. | There is no indication that all the vocabularies used should be fully FAIR compliant. In all the data related indicators for FHIR there is an assumption of there being a FHIR server with the data on it. | 1 |
| I3-01M | 1 | Profiles contain reference to, for example, data type descriptions or parent profiles. | 1 |  |  | 1 |
| I3-01D | 0 | Resource references. | 1 | Only if there is a FHIR server. |  | 1 |
| I3-02M | 1 | The implementation guide includes (references to) MIMIC-IV data | 1 |  |  | 1 |
| I3-02D | 0 | Resource references are qualified. | 1 | Only if there is a FHIR server. |  | 1 |
| I3-03M | 1 | Yes, for example the references to parent profiles. | 1 |  |  | 1 |
| I3-04M | 0 | No qualified references to other data (MIMIC-IV is linked but the relationship is not specified). After discussion: yes, for example references in the CapabilityStatement or implementation guide to other data sources. | 1 |  |  | 1 |
| R1-01M | 1 | Follows the FHIR specification and resource profiles. | 1 |  |  | 1 |
| R1.1-01M | 0 | License information is not included in the FHIR version of MIMIC-ED. | 0 |  |  | 0 |
| R1.1-02M | 0 | License information is not included in the FHIR version of MIMIC-ED. | 0 |  |  | 0 |
| R1.1-03M | 0 | License information is not included in the FHIR version of MIMIC-ED. | 0 |  |  | 0 |
| R1.2-01M | 0 | Provenance information is not included in the FHIR version of MIMIC-ED. | 1 | Only if there is a FHIR server. FHIR is a community specific standard. | The resources described in the implementation guide do not sufficiently capture provenance information. | 0 |
| R1.2-02M | 0 | Provenance information is not included in the FHIR version of MIMIC-ED. | 0 | Only if there is a FHIR server. The data does not contain this information |  | 0 |
| R1.3-01M | 1 | FHIR + profiles (US core and MIMIC). | 1 |  |  | 1 |
| R1.3-01D | 1 | FHIR | 1 |  |  | 1 |
| R1.3-02M | 1 | The FHIR specification (resource descriptions) is understandable by machines. | 1 |  |  | 1 |
| R1.3-02D | 1 | FHIR | 1 |  |  | 1 |
